# The Interplay of Ascorbic Acid with Quinones-Chelators—Influence on Lipid Peroxidation: Insight into Anticancer Activity

**DOI:** 10.3390/antiox11020376

**Published:** 2022-02-13

**Authors:** Olga Yu. Selyutina, Polina A. Kononova, Vladimir E. Koshman, Lidiya G. Fedenok, Nikolay E. Polyakov

**Affiliations:** Institute of Chemical Kinetics and Combustion, Institutskaya St., 3, 630090 Novosibirsk, Russia; kononova_polina@bk.ru (P.A.K.); kosmanvova2010@mail.ru (V.E.K.); fedenok@kinetics.nsc.ru (L.G.F.); polyakov@kinetics.nsc.ru (N.E.P.)

**Keywords:** anthraquinones, ascorbic acid, lipid peroxidation, anticancer activity, linoleic acid, NMR

## Abstract

Ascorbic acid is a multifaceted compound that can perform both antioxidant and pro-oxidant activities in the redox reactions induced by transition metal ions, so its role in nature and especially in the human body is still the subject of debate. In the present study, we have examined the influence of ascorbic acid on lipid peroxidation in a model system that mimics the cell membrane, namely micelles of linoleic acid (LA), induced by chelate complexes of iron and copper ions with quinone-chelator 2-phenyl-4-(butylamino)-naphtholquinoline-7,12-dione (Q1). This quinone effectively generates reactive oxygen species and semiquinone radicals inside cancer cells via a cycling redox reaction. Here it was demonstrated that in the absence of quinone-chelator ascorbic acid significantly accelerates the lipid peroxidation induced by both Fe(II) and Cu(II) ions. It has been shown also that Q1 chelate complexes with Fe(II) and Cu(II) ions are redox active in the LA micelles oxidation. No effect of ascorbate was detected on the reactivity of chelate complex with Fe(II) ions. On the other hand, ascorbate performs pro-oxidant activity in Q1-Cu(II) complex induced reaction. We can conclude that ascorbate-driven redox cycling of Q1 may promote its anti-tumor activity.

## 1. Introduction

Vitamin C or ascorbic acid (AA) is the most common vitamin, which is widely used as a nutraceutical supplement throughout the world [1,2]. AA is one of the most important plant-derived nutrients in the human diet found in most fruits and vegetables. Dietary AA is absorbed in the gastrointestinal tract and circulates in the blood in a free form [3]. AA is a hydrophilic compound and slowly penetrates through the cell membrane via passive transport, and the main pathway of AA penetration into the cell is regulated by sodium-dependent vitamin C transporter 1 and 2 (SVCT1 and SVCT2) [3,4]. The bioavailability of ascorbic acid is dose-dependent (higher absorption is observed at a lower dose) and is actively transported through cell membranes [5]. The concentration of vitamin C in plasma varies in the range ~50–150 μM. Intracellular vitamin C levels can reach significantly higher concentrations, up to 4 mM in lymphocytes or 10 mM in neurons [5]. Studies on different cell cultures revealed a high level of AA in mitochondria isolated from various animal tissues [3,6]. It is interesting from the point of antioxidant/pro-oxidant activity of AA due to the intensive production of reactive oxygen species in mitochondria [3,6].

In addition to its nutritional significance, AA is also used in medical practice and in clinical trials for the treatment of many diseases including hypovitaminosis C, severe pneumonia, severe acute respiratory failure, coronary artery disease, Type 2 diabetes, dementia, Alzheimer’s disease, and COVID-19 [7,8,9,10,11,12,13]. However, the benefit of using this dietary antioxidant in prophylactics and the treatment of cancer has been under debate for several decades already [14,15,16,17,18,19,20,21,22,23,24,25,26]. A number of clinical trials do not provide any reliable information. The studies showed either no effect of AA or a positive trend, although this has rarely been statistically proven in group comparisons [27].

**Figure 1:**
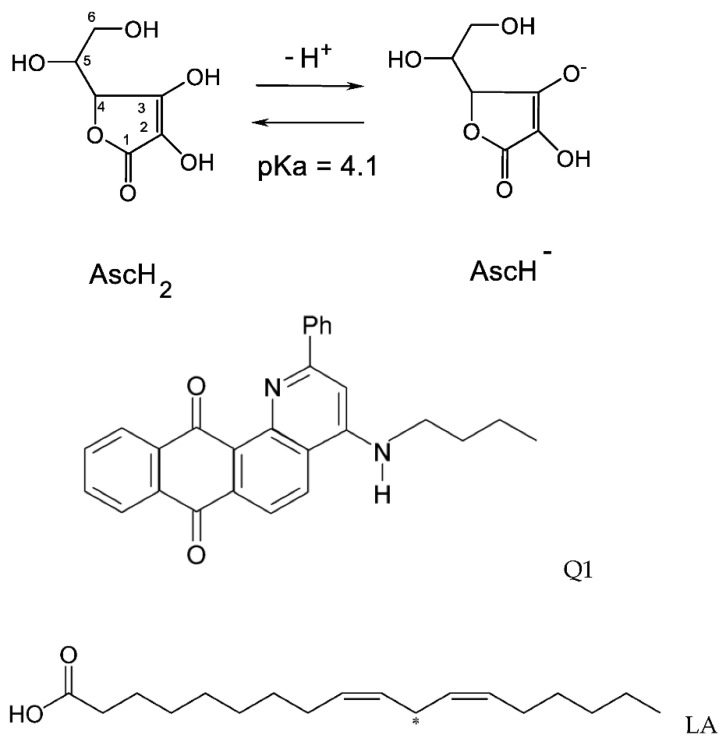
Structures of ascorbic acid (AscH2), quinone-chelator Q1: (2-phenyl- 4-(butylamino)naphtho[2,3-h]quinoline- 7,12-dione)), and linoleic acid (LA).

According to numerous literature data, AA is a multifaceted compound, and its role in nature and especially in the human body is still the subject of many experimental studies (see review [28] and refs therein). On the one hand, AA performs biological functions as a reducing agent and coenzyme in certain metabolic processes and also serves as an antioxidant. On the other hand, it demonstrates dual activity (antioxidant and pro-oxidant) in the redox reactions induced by transition metal ions, namely Cu and Fe [28,29,30,31,32,33]. The nature of the pro-oxidant activity of AA is related to its ability to reduce Fe(III) or Cu(II) ions via chelate complex formation, which is followed by the formation of corresponding Fe(II) and Cu(I) ions and ascorbic radical. In contrast to strong chelating drugs used in metal overload diseases for removing excess metals, AA is generally considered a weak chelating agent and cannot form strong metal complexes or be used effectively in metal removal. As a weak dibasic acid (pKa1 = 4.1 and pKa2 = 11.79), AA at physiological conditions exists as an ascorbate anion (AscH-) with a deprotonated 3-OH group at physiological conditions (see Figure 1). It was shown that the formation of chelate complexes of AA with metal ions occurs by chelation via O(3) and O(2) nuclei (Figure 1) [34].

**Scheme 1:**
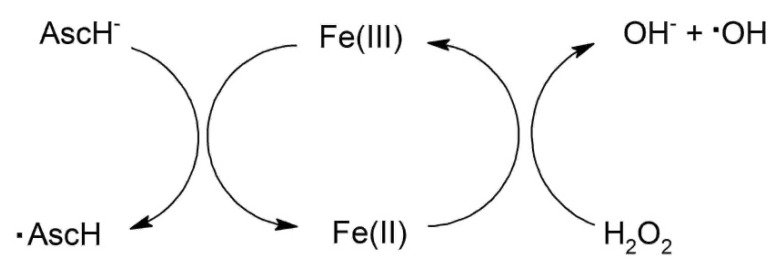
The AA-driven cyclic Fenton reaction. A schematic representation of the reduction of Fe(III) ion and the formation of hydroxyl radical in the presence of hydrogen peroxide.

The ability of AA to reduce ferric and cupric ions results in a significant increase in the yield of reactive oxygen species (ROS) in metal-induced redox reactions (Scheme 1) [35,36]. In particular, the increase of highly toxic hydroxyl radical (•OH) production in the Fenton reaction [37,38,39] has been demonstrated by the EPR spin trapping technique [29].

The thermodynamic rules for such redox-cycling were proposed that to be an effective catalyst, the metal-chelate complex should have a reduction potential between E°(O_2_/O_2_^•−^) = −0.33 V and E°(H_2_O_2_, H^+^/HO^•^, H_2_O) = 0.39 V [40].

According to literature, in vivo vitamin C may act more as a pro-oxidant than an antioxidant. Alternatively, vitamin C may successfully scavenge ROS. Vitamin C may affect the endogenous scavenging and repair systems, either directly, or indirectly. The ability of vitamin C to decrease the activity of endogenous antioxidant systems was reported in [41]. Experiments on mice revealed a significant reduction of expression of several genes linked to free-radical scavenging in the vitamin C-treated group.

Does the redox activity of AA matter in physiological conditions and should it be taken into account when studying the redox reactions of other chelators? It is known that in the human body under normal physiological conditions, there are very low concentrations of free, non-bound iron and copper [42,43]. In particular, Fe(III) ions are transported in blood by plasma transferrin, and stored intracellularly at low molecular weight iron pools composed of different natural chelators including AA and are utilized for the exchange of iron between the different sites of uptake, removal, and reuse [44,45]. Different mechanisms have been suggested regarding the role of AA in iron uptake and release from proteins including transferrin and ferritin [44,45,46]. However, the interaction of ascorbic acid with other redox-active drugs, and in particular with metal ion chelators, remains unexplored and at the same time very important field of science. The interaction of AA with transition metals is anticipated to affect the pharmacological and toxicological effects of other chelating drugs, the mechanism of which action is associated with iron or copper metabolism. In these regards, the co-administration of AA with iron or copper can affect the treatment of patients receiving anticancer drugs such as emodin, doxorubicin, etc. [47,48,49,50,51]. Furthermore, chelators such as deferiprone can be used as antidotes for preventing the pro-oxidant effects of AA [29,52,53].

The important field of metal-induced chemistry is lipid peroxidation. The widely used model molecule for lipid peroxidation study is linoleic acid (LA; Figure 1). The iron-induced peroxidation of linoleic acid is well characterized and involves the stages of ROS generation (Equation (1), Equations (1)–(4)); initiation of the reaction by hydrogen abstraction from LA (2); propagation with the formation of products in the form of hydroperoxides, aldehydes, and epoxides (3); and the termination stage (4).

Fe^2+^ + H_2_O_2_ → Fe^3+^ + OH^•^ + OH^−^ (1)

LH + OH^•^ → L^•^ + H_2_O (2)

L^•^ + O_2_ → LOO^•^

LOO^•^ + LH → LOOH + L^•^ (3)

LOOH → LO^•^ → epoxides, hydroperoxides, aldehydes

L^•^ + L^•^ → L-L (4)

LOO^•^ + L^•^ → LOOL

LOO^•^ + LOO^•^ → LOOL + O_2_

Equations (1)–(4): The Equations of iron-induced lipid (LH) peroxidation [54,55,56]. A similar mechanism can be suggested for the copper-induced Fenton reaction [39].

By changing the redox status of metal ions in Fenton-like processes, chelating drugs can affect (inhibit or accelerate) this reaction. The influence of ascorbate ions on this process in the absence of other chelating drugs is mediated by the reduction of Fe(III) to Fe(II):

AscH^−^ + Fe^3+^ → Asc^•−^ + Fe^2+^ + H^+^ (5)

The possibility of ascorbate oxidation by various chelate complexes, including complexes of anthraquinones containing chelating sites, and involvement of this reaction in redox cycling of ROS generation is still under debate and will be discussed in the Discussion section [28,51,57].

Substituted anthraquinones known as anthracycline antibiotics (doxorubicin, daunomycin, emodin, etc.) are widely used in cancer therapy [58]. Two mechanisms are proposed by which these quinones act in the cancer cell. First is the intercalation into DNA duplexes, and second is the generation of ROS which destroy the cellular membranes by stimulation of lipid peroxidation [59,60,61]. On the other hand, it is important that especially the damage of the cell membranes as a result of lipid peroxidation is commonly considered as the main mechanism of cardiotoxicity of some anthracycline anticancer compounds widely used in medical practice [48,49]. The mechanism of quinone-induced ROS production is shown in Equations (6)–(11). At the first stage, quinone can be reduced by natural reductases, namely nicotinamide adenine dinucleotide phosphate (NAD(P)H), ascorbic acid, or reduced glutathione, following the semiquinone radical production. Under aerobic conditions, semiquinone radical anions initiate the formation of ROS via the series of reactions shown in Equations (6)–(11) [62]. The reaction of ROS generation can be accelerated by the presence of trace amounts of transition-metal ions, usually iron ions (Equations (10) and (11)).

Q + e → Q^•−^ (6)

Q^•−^ + O_2_ ↔ Q + O_2_^•−^ (7)

O_2_^•−^ + O_2_^•−^ → H_2_O_2_ + O_2_ (8)

O_2_^•−^ + H_2_O_2_ → O_2_ + HO^−^ + HO^•^ (9)

Q^•−^ + Fe(III) → Q + Fe(II) (10)

H_2_O_2_ + Fe(II) → HO^−^ + HO^•^ + Fe(III) (11)

Equations (6)–(11): The Equations of ROS generation during the interaction of quinones (Q) with electron donors in the presence of iron in the system.

Note that the reactions shown in Equations (6)–(11) are valid only for quinones possessing redox potentials that permit them to oxidize the above-mentioned reductants, and then go on to reduce molecular oxygen and Fe(III) ions. The important feature of anthraquinones that demonstrate iron ions-sensitive ROS generation ability (Doxorubicin, Daunomycin, Emodin), is the presence of an iron-chelating site, namely, the 1-OH group near the carboxyl group. It was proposed that the presence in the same chelate complex of both a quinone center and a redox-active metal ion, can substantially enhance the rate of ROS generation, and thus of cell death [47,48,49,63].

In this study we focus our attention on the dual effect on lipid peroxidation of AA and another metal-binding ligand that possess anti-cancer activity, namely 2-phenyl-4-(butylamino)naphtho[2,3-h]quinoline-7,12-dione), (Q1; Figure 1) [47,64]. In contrast to 1-OH substituted anthraquinones like doxorubicin or emodin, the chelating site of Q1 consists of O and N atoms. Earlier it was demonstrated that quinone-chelator Q1 can be effectively reduced by AA, glutathione, and NADH with the formation of the free semiquinone radical as well as ROS [47,65]. Note, that for some cell lines Q1 showed higher activity in ROS generation than Doxorubicin [66]. For example, Q1 activity towards breast cancer cells MFC-7 exceed doxorubicin activity more than 100 times. It is important to note that quinone with similar redox potential as Q1, but without the chelating center, shows much lower ROS generating ability and no activity towards cancer cells [47,64]. The reason for such effect is a significant influence of chelation on the redox potential of quinone [47,63].

The current investigation examined the influence of the iron and copper complexes of quinone Q1 on lipid peroxidation in a model system that mimics the cell membrane, namely micelles of linoleic acid. In addition, the role of AA in the mechanism of ROS generation was elucidated.

## 2. Materials and Methods

### 2.1. Materials

Quinone-chelator, 2-phenyl-4-(butylamino)naphtho[2,3-h] quinoline-7,12-dione (Q1, Figure 1) was synthesized according procedure described by Dikalov et al. [63]. Linoleic acid > 99.0% purity was purchased from Shanghai Aladdin Bio-Chem Technology Co., Ltd., Shanghai, China. Deuterated water D_2_O (99.9% D), Ascorbic acid (99%), FeSO_4_, CuCl_2_, and H_2_O_2_ (35.5%) were obtained from Sigma Aldrich.

### 2.2. Sample Preparation for Lipid Peroxidation Studies

Reaction mixtures for the ^1^H NMR studies consisted of LA micelles (3.5 mM of LA). In reactions with hydrogen peroxide, 0.5 M H_2_O_2_ was added with 0.1 mM of FeSO_4_ or CuCl_2_ freshly prepared in PBS (pH 7.4). For studies examining the effects of Q1 on LA oxidation via the Fenton reaction, quinone was added to the lipid solution in chloroform before drying and removal of the solvent and subsequent hydration in PBS (pH 7.4). The final concentration of ligand was 0.2 mM.

For studies examining the effect of ascorbate, a final concentration of 2.5 mM was utilized. The ^1^H NMR spectra were recorded on a Bruker AVHD-500 (500 MHz) NMR spectrometer (Bruker, Billerica, MA, USA) using a temperature of 298 K.

### 2.3. UV-Vis Spectrophotometry

Measurements of optical absorption spectra were performed using an SF-2000 spectrophotometer (Spectrum, Moscow, Russia) in a 1 cm quartz cuvette.

### 2.4. The Q1-Cu(II) Complex Stoichiometry and Stability Calculations

Calculation of chelate complex parameters was carried out on the basis of the following model of complex formation [53,67]:

L + M ↔ LM(12)

L + LM ↔ L2M(13)

The method of calculation of stability constants K_1_ and K_2_ and extinction coefficients ε_1_ and ε_2_ for 1:1 and 2:1 complexes, respectively, is described in [53,67]. Briefly, the dependencies of the relative change of the ligand absorption (∆D) on Cu(II) concentration were obtained. The analytical expression for ∆D in this model is [53,67]:

∆D = ε_0_[L] + ε_1_K_1_[L][M] + ε_2_ K_1_K_2_[L]^2^[M] − ε_0_C_L_, (14)

where ε_0_, ε_1,_ and ε_2_ are extinction coefficients of the ligand, the complex 1:1 and the complex 2:1, respectively, K_1_, K_2_ are stability constants of the complex 1:1 and the complex 2:1, respectively, C_L_ is the initial ligand concentration. The stability constants and extinction coefficients were obtained from fitting the parameters using the Levenberg–Marquardt algorithm via the specially designed Python script.

### 2.5. Statistical Analysis

For comparison of two experimental conditions, a two-tailed, unpaired Student’s *t*-test was performed using at least 3 independent experiments. Statistical significance was set at *p* < 0.05. Data are presented as mean ± standard deviation (number of experiments).

## 3. Results and Discussion

### 3.1. Cu(II) Complex Formation Study

**Figure 2:**
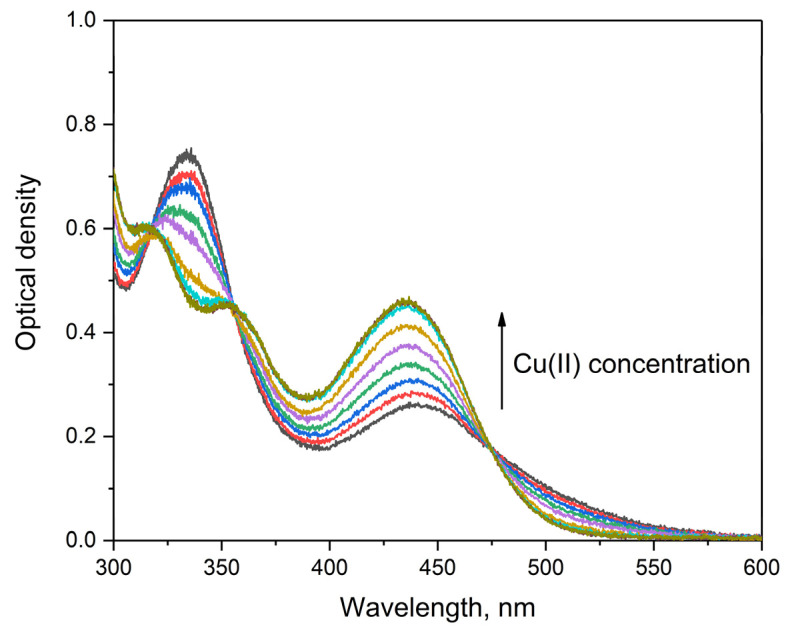
UV-Vis absorption spectra of 0.06 mM Q1 solutions containing CuCl_2_ in the concentration range 0–0.04 mM in ethanol at 25 °C.

It was described previously that Q1 could act as chelating agent and form complexes with Fe(II) and Fe(III) ions, so, in the present work, the possibility of only Cu(II)-Q1 complex formation was checked. The complex formation has been proved using optical spectroscopy. Optical absorption spectra of the quinone were measured for Cu(II) concentration ranging from 0.005 to 0.04 mM. Q1 concentration was 0.06 mM. Experiments were performed in ethanol and in a mixture of ethanol (80%) and water (20%). Optical absorption spectra of Cu(II)-Q1 in ethanol are presented in Figure 2. The same behavior was observed in the water–ethanol mixture.

**Figure 3:**
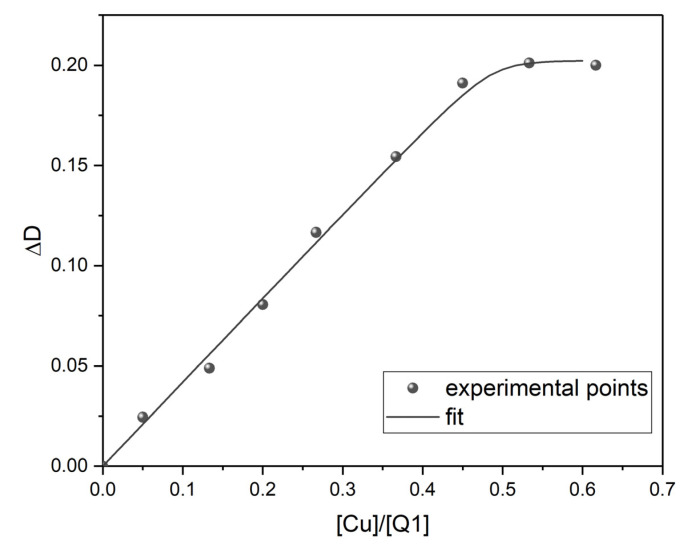
The dependence of the relative change of the ligand absorption (∆D) at 435 nm on Cu(II)/Q1 concentration ratio in ethanol.

The dependencies of the relative change of the ligand absorption (∆D) on Cu(II)/Q1 concentration ratio at 435 nm were obtained and fitted via expression (14) (See Section 2.4 for details). Experimental points and fitting curves for ethanol solution are presented in Figure 3. Similar results were obtained in the water–ethanol mixture.

The presence of isosbestic points and the saturation of ∆D at the metal–ligand ratio 0.5 indicates that the complex has the stoichiometry 1:2 (metal:ligand). The complex stability constants and extinction coefficients obtained from the fit are presented in Table 1.

According to our previous study K_1_ = (4.4 ± 1.0) × 10^5^ M^−1^ and K_1_ = (6.2 ± 2.1) × 10^5^ M^−1^ for Fe(II)-Q1 chelate complex [67]. We can see that complex Cu(II)-Q1 in ethanol is more stable than Fe(II)-Q1 complex.

### 3.2. Iron-Mediated Peroxidation of LA Micelles in the Presence of H_2_O_2_

**Figure 4:**
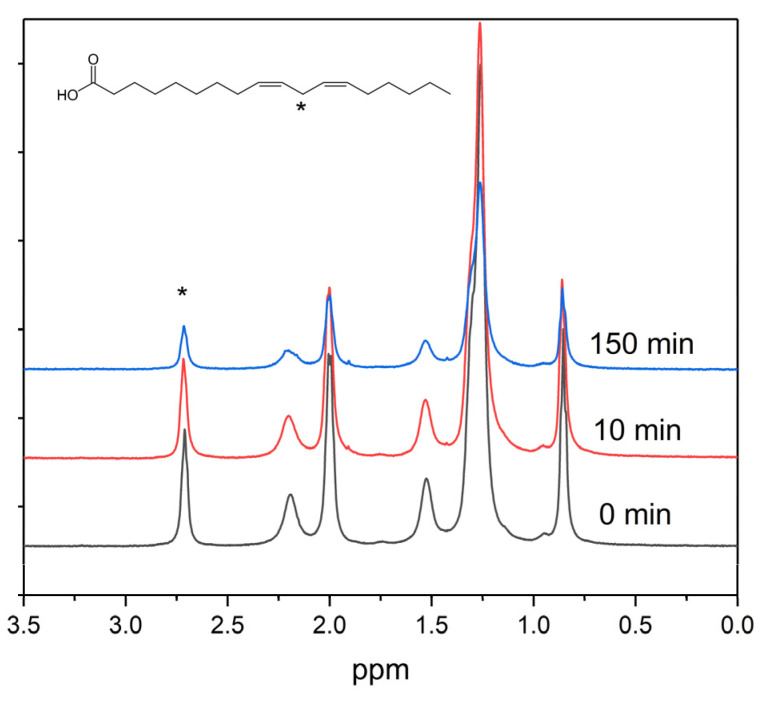
The fragments of ^1^H NMR spectra of the initial LA solution (3.5 mM), and at different delay times after the start of the reaction. The signal marked with (*) corresponds to bis-allylic protons of LA.

**Figure 5:**
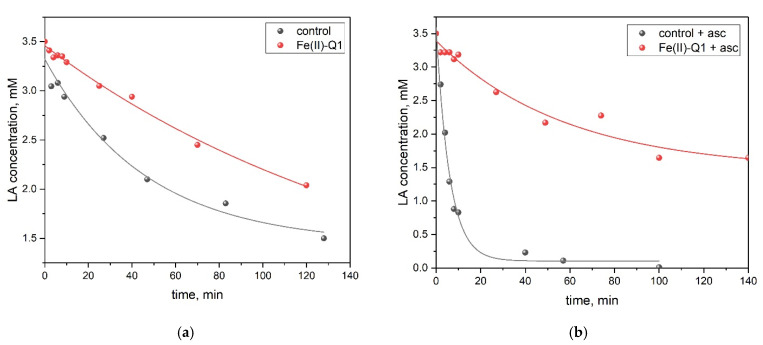
Kinetics of the initiation reaction of LA peroxidation in the absence and presence of the iron complexes of Q1 (see Materials and Methods for details), either: (**a**) without ascorbate, (**b**) with 2.5 mM ascorbate at 25 °C. Points-experiment, solid line-exponential fitting.

Lipid micelles are a common model mimicking the biological membrane properties [68]. LA is an example of a polyunsaturated fatty acid where the peroxidation is well described [68,69]. In the present study, examining the peroxidation of LA (3.5 mM) was performed in the presence of H_2_O_2_ (0.5 M) and FeSO_4_ (0.1 mM) at 25 °C (pH 7.4). The analysis of the kinetic of LA peroxidation was performed with the approach described in [70]. Briefly, the time dependence of the intensity of the NMR signal of bis-allylic protons of LA (Figure 4, 2.7 ppm, asterisk) was measured. Since the initiation stage of LA oxidation is the abstraction of a hydrogen atom at this position, and reaction products (lipid radicals and conjugated dienes, Equations (1)–(4)) do not contain such protons in the structure, the initiation stage leads to a decrease in the intensity of this signal as a function of time (Figure 5). Control experiments using the mixture of LA + Fe(II) in the absence of H_2_O_2_ demonstrated no decrease in the intensity of LA signals in the spectrum during 24 h at 25 °C.

The kinetics of LA (3.5 mM) peroxidation at pH 7.4 in the reaction with H_2_O_2_ (0.5 M)/25 °C were measured for Fe(II) (as FeSO_4_; 0.1 mM) and for iron complex of Q1 (0.1 mM, i.e., [ligand] = 0.2 mM and [Fe] = 0.1 mM), in the absence (Figure 5a) or presence (Figure 5b) of ascorbate (2.5 mM). The experimental points were approximated by an exponential decay, and the first-order reaction rate constants have been obtained from fitting the parameters using the Levenberg–Marquardt algorithm. The calculated reaction rate constants are given in Table 2.

In the absence of Q1, the observed oxidation of LA was consistent with Equations (1)–(4). Taking into account that the reaction proceeds in an excess of H_2_O_2_, the fast conversion of Fe(II) to Fe(III) occurs within a millisecond time scale in the first stage due to the reaction in Equation (1). The rate constant of this reaction is 63 M^−1^s^−1^ [37,38]. We assume that the limiting stage of LA oxidation is the conversion of Fe(III) to Fe(II) according to the reaction in Equation (15) below, which proceeds with a rate constant of 0.0027 M^−1^s^−1^ [37,38].

Fe^3+^ + H_2_O_2_ → Fe^2+^ + OOH^•^ + H^+^ (15)

In the absence of ascorbate, a rate constant of LA peroxidation induced by Fe(II)-Q1 is 3.6-fold lower (*p* < 0.05) than induced by unchelated Fe(II) control (Table 2). The addition of ascorbate to the reaction system contained unchelated Fe(II) (as FeSO_4_) increased the rate constant of LA peroxidation by 7.5-fold versus the analogous condition in the absence of ascorbate (Table 1; Figure 5a,b). This observation is probably due to the fast transformation of Fe(III) to Fe(II) in the presence of ascorbate (Equation (5)). The addition of ascorbate to the Fe(II)-Q1 complex leads to an insignificant increase of the rate constant of LA peroxidation (Table 2) relative to Fe(II)-Q1 in the absence of ascorbate (Figure 5b).

In the presence of ascorbate and the iron complex of Q1, the rate constant of LA peroxidation is significantly lower than that for unchelated Fe(II) control (Figure 5b). This observation demonstrates that Fe(II) reacts more readily to induce LA peroxidation when it is “free” relative to when chelated by Q1. This latter observation can be explained by the fact that Q1 binds iron and prevents its reaction with ascorbate, and thus acts as an antioxidant compared to unchelated iron.

In summary, the results shown in Figure 5 and Table 2, show that Fe(II)-Q1 complex acts as an antioxidant, reducing LA peroxidation compared to the Fe(II) control.

### 3.3. Copper-Mediated Peroxidation of LA Micelles in the Presence of H_2_O_2_

**Figure 6:**
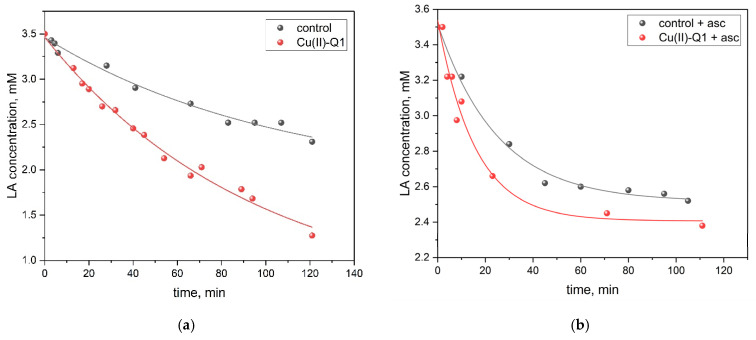
Kinetics of the initiation reaction of LA peroxidation in the absence and presence of the copper complexes of Q1 (see Materials and Methods for details), either: (**a**) without ascorbate, (**b**) with 2.5 mM ascorbate at 25 °C.

The kinetics of copper-induced LA peroxidation at pH 7.4 in the reaction with H_2_O_2_ were measured in the absence (Figure 6a) or presence (Figure 6b) of ascorbate in the same way as described above (see Materials and methods and Section 3.1 for the details). The calculated reaction rate constants are given in Table 3.

In the absence of ascorbate, a rate constant of LA peroxidation induced by Cu(II)-Q1 is 1.6-fold higher (*p* < 0.05) than unchelated Cu(II) control (Table 3). The addition of ascorbate and unchelated Cu(II) (as CuCl_2_) increased the rate constant of LA peroxidation by 8.2-fold compared to the analogous condition in the absence of ascorbate (Table 3; Figure 6a,b). This observation is probably due to the fast transformation of Cu(II) to Cu(I) in the presence of ascorbate (Equation (18)). The addition of ascorbate to the Cu(II)-Q1 complex leads to a significant increase of the rate constant of LA peroxidation (Table 3) relative to Cu(II)-Q1 in the absence of ascorbate (Figure 6).

According to literature data, Cu(II) reacts with H_2_O_2_ by following mechanism [53,71,72,73,74]:

Cu(II) + H_2_O_2_ → Cu(I) + O_2_^•−^, (16)

Cu(I) + H_2_O_2_ → Cu(II) + OH^•^ + OH^−^, (17)

ROS produced in these reactions lead to the formation of the hydroperoxide LOOH (Equations (1)–(4)).

Ascorbic acid is known to increase the yield of OH^•^ by reduction of Cu(II) to Cu(I) [75]:

AscH^−^ + Cu(II) → Asc^•−^ + Cu(I) + H^+^ (18)

The attenuation of the copper-induced lipid peroxidation in the presence of ascorbate is described in the literature [76].

In the presence of ascorbate and the copper complex of Q1, the rate constant of LA peroxidation is significantly higher than that of the unchelated Cu(II) control (Figure 4b).

**Scheme 2:**
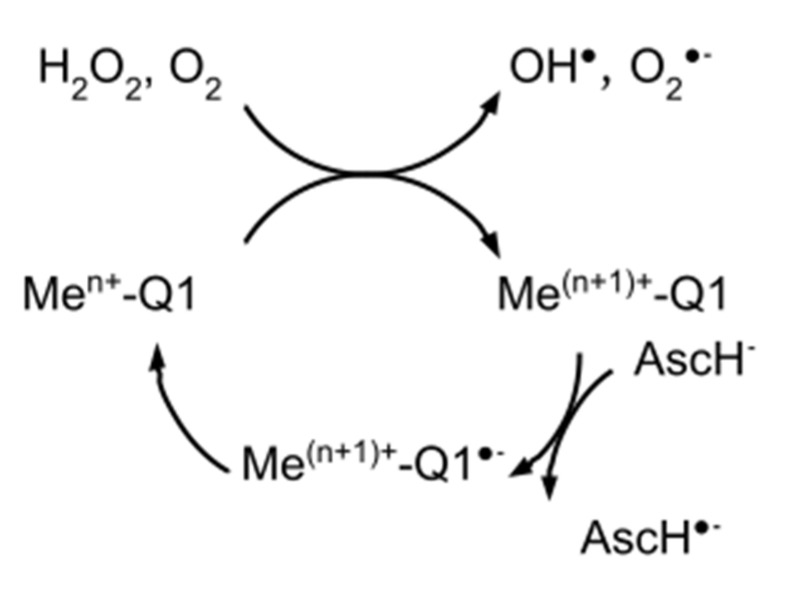
The scheme of ROS generation during the interaction of quinone Q1 with electron donors in the presence of metal ions (Me) and ascorbate in the system.

In summary, the results shown in Figure 6 and Table 3 indicate that Cu(II)-Q1 complex acts as a pro-oxidant by increasing the initiation rate of LA peroxidation versus the Cu(II) control. We assume that the observed difference in ascorbate effect on LA peroxidation induced by iron and copper chelate complexes with Q1 is due to the difference in rate constants of electron transfer from ascorbate to Cu(II)-Q1 and Fe(III)-Q1 complexes (see Scheme 2). The possibility of such reaction for Fe(III)-Q1 complex was earlier reported by Dikalov, et al. [65].

## 4. Conclusions

In the present study, we have examined the influence of ascorbic acid on lipid peroxidation in a model system that mimics the cell membrane, namely micelles of linoleic acid, induced by chelate complexes of iron and copper ions in the presence of hydrogen peroxide. For direct calculation of the rate constant of the initiation stage of lipid peroxidation, we used an original approach based on the measurement of the intensity of the NMR signal of LA bis-allylic proton [70]. Another methodological approach we used in this study to model oxidative stress is the application of chelate complexes of quinone 2-phenyl-4-(butylamino)-naphtholquinoline-7,12-dione for the generation of ROS. According to previous studies, this quinone-chelator is reduced enzymatically, inside the cells, by NAD(P)H dependent enzymes or reduced glutathione, and effectively generates ROS inside cancer cells via cycling redox reaction [47,64]. Since the significant concentrations of iron and copper ions and biological reducing agents are found exclusively inside the cell one can be sure that ROS production occurs only within the cell and does not originate outside of the cell. Cuprum ions play an important role in ROS generation in lysosomes [77,78].

As was earlier shown by EPR spin trapping, ascorbate anions demonstrate pro-oxidant activity in Fenton-like reactions in homogeneous solutions in the absence of metal chelators [28,29]. Here it was demonstrated that in the absence of quinone-chelator ascorbate also significantly accelerates the LA micelles oxidation induced by both Fe(II) and Cu(II) ions.

It has been shown also that Q1 chelate complexes with Fe(II) and Cu(II) ions are redox-active in the LA micelles oxidation. The corresponding initiation rate constants are 1.0 ± 0.40 × 10^−4^ s^−1^ and 1.3 ± 0.30 × 10^−4^ s^−1^. No effect of ascorbate was detected on the reactivity of the chelate complex with Fe(II) ions. On the other hand, Fe(II)-Q1 complex formation in the presence of ascorbate significantly reduces the peroxidation rate compared with unbounded iron ions. This observation might be important from the point of view of the reduced cardiotoxicity of this chelate complex in cancer therapy. On the contrary, ascorbate performs pro-oxidant activity in a Q1-Cu(II) complex induced reaction. The calculated initiation rate constant of ascorbate-driven LA peroxidation is 9.0 ± 0.40 × 10^−4^ s^−1^.

Obtained results may help in understanding the possibility of using ascorbic acid for tumor therapy. A number of studies have shown that pharmacological concentrations of ascorbic acid selectively kill cancer cells in vitro (see, for example, review [79]). In our previous works the antiproliferative activity of ascorbic acid on the human melanoma cell line, SK-MEL-28 was studied [80]. No antiproliferative activity of ascorbic acid alone was noted, but the high activity of the combination of ascorbic acid with two different chelators was observed. Note that to treat cancer, redox-active chelators are required, which enhance the redox properties of bounded metal ions. In addition, the chelating and reducing properties of ascorbic acid open the possibility of its use together with anticancer chelating agents in complex therapy [81].

## Figures and Tables

**Table 1:** Calculated parameters of Q1 chelating complexes in ethanol solution and in water–ethanol mixture.

	Cu(II)-Q1 in Ethanol	Cu(II)-Q1 in 80% Ethanol
*ε*_1_	(0.7 ± 0.01) × 10^4^	(1.2 ± 0.03) × 10^4^
*K*_1_	(1 ± 0.6) × 10^6^ M^−1^	(5 ± 0.7) × 10^5^ M^−1^
*ε*_2_	(1.56 ± 0.02) × 10^4^	(1.47 ± 0.01) × 10^4^
*K*_2_	(5 ± 0.7) × 10^6^ M^−1^	(5 ± 0.8) ×10^6^ M^−1^

**Table 2:** Rate constants for the initiation reaction of LA peroxidation in micelles induced by the Q1 iron complexes relative to the control (i.e., unchelated Fe(II) added as FeSO_4_) in the presence and absence of ascorbate (2.5 mM).

	Control (Fe(II))	Fe(II)-Q1
(−) ascorbate	3.60 ± 0.80 × 10^−4^ s^−1^	1.0 ± 0.40 × 10^−4^ s^−1^
(+) ascorbate	27.00 ± 0.40 ×10^−4^ s^−1^	1.2 ± 0.60 × 10^−4^ s^−1^

**Table 3:** Rate constants for the initiation reaction of LA peroxidation in micelles induced by the Q1 copper complexes relative to the control (i.e., unchelated Cu(II) added as CuCl2) in the presence and absence of ascorbate (2.5 mM).

	Control (Cu(II))	Cu(II)-Q1
(−) ascorbate	0.8 ± 0.20 × 10^−4^ s^−1^	1.30 ± 0.30 × 10^−4^ s^−1^
(+) ascorbate	6.6 ± 0.4 × 10^−4^ s^−1^	9.0 ± 0.40 × 10^−4^ s^−1^

## Data Availability

Data is contained within the article.
